# Supplementary material for: Sexual inhibition and sexual excitation in a sample of Polish women
Source: PLoS One. 2021 Apr 6;16(4):e0249560. doi: 10.1371/journal.pone.0249560 (PMC8023475; doi:10.1371/journal.pone.0249560)
Supplement: S1 Table — (DOCX) [file pone.0249560.s001.docx]

Table 6. SESIIW-PL correlations with other measurement (based on Pearson’s R statistics)

| **Measurement** | **Arousability** | **Partner Characteristics** | **Smell** | **Sexual Power Dynamics** | **Relationship Importance** | **Concerns about Sexual Function** | **Arousal Contingency** | **SES** | **SIS** |
| --- | --- | --- | --- | --- | --- | --- | --- | --- | --- |
| **Age** | -.34** | -.35** | -.15** | -.46** | -.11* | -.16** | 0.03 | -.41** | -.10* |
| **BMI** | -.07 | -0.11 | -0.01 | -0.08 | 0.03 | -0.07 | 0.07 | -0.08 | 0.01 |
| **Being in relationship** | .01 | -,15** | -.03 | -.04 | .15** | .05 | .04 | -.07 | .11* |
| **Nr of sexual partners** | .09* | .28** | .11* | .21** | -.14** | -0.02 | -0.01 | .22** | -0.08 |
| **Nr of vaginal sex/month** | .22** | .13** | .11* | .15** | 0.03 | -0.06 | -.17** | .19** | -.09* |
| **Masturbation masturbation/month** | .22** | .13** | .15** | .17** | 0.04 | -0.05 | -.16** | .21** | -0.08 |
| **Sefl masturbation/month** | .20** | .26** | .15** | .28** | 0.02 | 0.03 | -0.03 | .29** | 0.01 |
| **RSB** | .11** | .21** | 0.07 | .18** | -.25** | -0.04 | -0.05 | .18** | -.14** |
| **HADS-A – score** | -0.06 | 0.00 | 0.05 | -0.02 | 0.05 | .17** | .21** | -0.01 | .19** |
| **HADS-D - score** | -.13** | -0.03 | -0.03 | -0.03 | -0.01 | .16** | .22** | -0.07 | .17** |
| **HADS-A - YES** | 0.00 | 0.03 | 0.05 | 0.01 | 0.06 | 0.05 | .12** | 0.03 | .10* |
| **HADS_D - YES** | 0.00 | 0.08 | 0.01 | 0.03 | -0.02 | 0.09 | 0.06 | 0.04 | 0.06 |
| **FSFI score** | .29** | 0.08 | .14* | 0.11 | 0.03 | -.20** | -.42** | .20** | -.28** |
| **Sexual problems (FSFI score) - YES** | -.23** | -0.01 | -0.10 | -0.03 | -0.06 | .24** | .38** | -0.12 | .27** |
| **FSDS-R score** | -.21** | 0.04 | -0.04 | 0.02 | -0.01 | .29** | .42** | -0.05 | .33** |
| **Distress (based on FSDS-R) – YES** | -.17** | 0.03 | -0.04 | -0.01 | -0.08 | .15** | .34** | -0.06 | .20** |
| **Distressing sexual problems (FSFI + FSDS-R) - YES** | -.21** | -0.01 | -0.05 | -0.05 | -0.04 | .20** | .33** | -0.10 | .23** |
| **FSD – DSM-5 - YES** | -0.03 | 0.09 | 0.06 | 0.06 | 0.00 | .12** | 0.08 | 0.06 | .09* |
| **BAS-D** | 0.09 | 0.08 | 0.09 | 0.01 | -0.09 | -0.07 | 0.03 | 0.09 | -0.05 |
| **BAS-FS** | .17** | .23** | 0.12 | .22** | -.21** | -0.11 | -0.05 | .25** | -.16** |
| **BAS-RR** | .14* | .13* | 0.03 | 0.11 | -0.06 | -0.03 | -0.01 | .13* | -0.04 |
| **BIS** | 0.01 | 0.05 | 0.01 | 0.04 | 0.12 | .22** | .18** | 0.04 | .23** |
| **SOS-SF** | -.29** | -.39** | -.17** | -.41** | .35** | 0.02 | 0.06 | -.42** | .17** |
| **SOI-R** | .17** | .54** | .22** | .23** | -.41** | -0.05 | 0.00 | .40** | -.18** |
| **SSSS** | .26** | .40** | .22** | .32** | -.17** | -0.05 | 0.01 | .41** | -0.09 |
| **SDQ** | 0.08 | 0.01 | 0.02 | -0.01 | 0.01 | 0.06 | -0.02 | 0.02 | 0.02 |
| **TITP - E** | .16* | .14* | 0.09 | 0.08 | -0.03 | -.14* | -.26** | .15* | -.19** |
| **TIPI - A** | .18** | 0.00 | 0.12 | 0.01 | 0.05 | -0.04 | -.17** | 0.10 | -0.08 |
| **TIPT - C** | 0.10 | 0.03 | 0.05 | -0.01 | 0.09 | -0.07 | -.13* | 0.05 | -0.06 |
| **TIPI - N** | 0.01 | -0.07 | -0.04 | -0.03 | -.14* | -.22** | -.27** | -0.05 | -.28** |
| **TIPI - OTE** | 0.08 | .16* | 0.02 | .20** | -.14* | 0.00 | -.20** | .15* | -.15* |
| **WMRQ - I** | -.16** | -.18** | 0.05 | -.21** | -0.11 | -.11* | 0.01 | -.14** | -0.09 |
| **WMRQ - D** | -.14* | 0.02 | -.14* | -0.03 | -.21** | 0.05 | .13* | -0.10 | -0.01 |
| **WMRQ - PF** | -.22** | -.19** | -0.01 | -.28** | -0.08 | -0.05 | 0.03 | -.22** | -0.04 |
| **WMRQ - S** | -.14** | -.19** | 0.05 | -.21** | -0.07 | -0.07 | 0.01 | -.15** | -0.06 |
| **WMRQ - total** | -.12* | -.17** | 0.07 | -.20** | -0.02 | -0.09 | -0.02 | -.13* | -0.06 |

* - p<.05; ** - p<.001; BMI – body mass index; RBS – risky sexual behaviors; HADS – Hospital Anxiety and Depression Scale; HADS-D – depression mode, HADS-A – anxiety mode; FSD - Female Sexual Dysfunction; FSFI – Female Sexual Function Index; FSDS-R - Female Sexual Distress Scale-Revised; DSM-5 - Diagnostic and Statistical Manual of Mental Disorders, 5^th^ Edition; BAS-D - Behavioural Activation Scale – drive; BAS-FS - fun seeking; BAS-RR - Behavioural Activation Scale - reward responsiveness; BIS - Behavioural Inhibition Scale; SOS-SF - Sexual Opinion Survey – Short Form; SOI-R - Sociosexual Orientation Inventory Revised; SSSS - Sexual Sensation Seeking Scale; SDQ - Social Desirability Questionnaire. TIPI - Ten-Item-Personality-Inventory; TITP-E – Extraversion; TIPI-A – Agreeableness; TIPT-C – Conscientiousness; TIPI-N – Neuroticism; TIPI-OTE - Openness to Experience; WMRQ-I – Well-Matched Relationship Questioner – intimacy; WMRQ-D - Well-Matched Relationship Questioner – disappointment; WMRQ-PF - Well-Matched Relationship Questioner - personal fulfilment; WMRQ-S - Well-Matched Relationship Questioner – similarity; WMRQ - total - Well-Matched Relationship Questioner – total score; SES - Sexual Excitation Scale; SES-A – Arousability; SES-PC - Partner Characteristics; SES-SPD - Sexual Power Dynamics; SES-S – Smell; SES-Set - Setting (Unusual or Unconcealed); SIS - Sexual Inhibition Scale ; SIS-CSF - Concerns about Sexual Function; SIS-AC - Arousal Contingency; SES-RI - Relationship Importance.
