# Supplementary material for: Sexual inhibition and sexual excitation in a sample of Polish women
Source: PLoS One. 2021 Apr 6;16(4):e0249560. doi: 10.1371/journal.pone.0249560 (PMC8023475; doi:10.1371/journal.pone.0249560)
Supplement: S1 Appendix — (DOCX) [file pone.0249560.s002.docx]

**Kwestionariusz pobudzenia i hamowania seksualnego – wersja dla kobiet**

**Instrukcja**

Poniższy kwestionariusz ma za zadanie wskazać rzeczy, które mają wpływ na poziom Twojego podniecenia seksualnego. Kobiety opisują swoje podniecenie seksualne w bardzo różny sposób. Opisy te mogą dotyczyć zmian zachodzących w kobiecych narządach płciowych (jestem „wilgotna”, czuję „mrowienie”, czuję się „rozpalona”, jak i mogą być związane z nie-genitalnymi odczuciami (przyspieszenie akcji serca, zmiana temperatury, wrażliwość skóry, itp.), lub emocjami (oczekiwanie, niecierpliwe wyczekiwanie, poczucie bycia „seksownym” albo „seksualnym, itp.).

Jesteśmy zainteresowani jakie są Twoje typowe reakcje na poniższe stwierdzenia. Niektóre
z tych stwierdzeń mogą Cienie nie dotyczyć lub mogły Ciebie dotyczyć w przeszłości, a teraz już nie. W takich sytuacjach proszę odpowiedzieć na te stwierdzenia tak, jakbyś postąpiła, gdybyś rzeczywiście znalazła się w takich okolicznościach. Niektóre z poniższych stwierdzeń brzmią bardzo podobnie, natomiast mają inne znaczenia.

Proszę przeczytaj uważnie każde z poniższych zdań, a następnie zaznacz kółkiem odpowiedź, która wyraża Twoją opinię. Nie zastanawiaj się długa nad odpowiedziami. Proszę zaznacz odpowiedź, która pierwsza przyszła Ci do głowy.

|  | **Stwierdzenia:** | **Całkowicie się nie zgadzam.** | **Raczej się nie zgadzam.** | **Raczej się zgadzam.** | **Całkowicie się zgadzam.** |
| --- | --- | --- | --- | --- | --- |
| 1. | Podnieca mnie, jak partner świntuszy podczas seksu. | 1 | 2 | 3 | 4 |
| 2. | Podnieca mnie seks w innych niż zazwyczaj miejscach. | 1 | 2 | 3 | 4 |
| 3. | Podniecają mnie osoby, które uchodzą za inteligentne | 1 | 2 | 3 | 4 |
| 4. | Poczucie bycia zdominowaną w sytuacji seksualnej przez osobę, której ufam, podnosi poziom mojego podniecenia. | 1 | 2 | 3 | 4 |
| 5. | Jeśli martwię się tym, czy jestem dobrą kochanką, trudniej mi się podniecić | 1 | 2 | 3 | 4 |
| 6. | Oglądanie partnera wykonującego czynności ukazujące jego talent wpływa na mnie bardzo podniecająco. | 1 | 2 | 3 | 4 |
| 7. | Ciężko by mi było podniecić się seksualnie, kiedy miałabym świadomość, że partner jest zaangażowany w relację z inną osobą. | 1 | 2 | 3 | 4 |
| 8. | Podnieca mnie kontakt wzrokowy z osobą, którą uważam za atrakcyjną seksualnie | 1 | 2 | 3 | 4 |
| 9. | Jeśli czuję, że ktoś mnie wykorzystuje seksualnie, podniecenia seksualne ustępuje natychmiast. | 1 | 2 | 3 | 4 |
| 10. | Podnieca mnie oglądanie nagiego ciała atrakcyjnego partnera seksualnego. | 1 | 2 | 3 | 4 |
| 11. | Łatwiej jest mi się podniecić przy osobie, która jest „materiałem na partnera” | 1 | 2 | 3 | 4 |
| 12. | Samo bliskość fizyczna partnera wystarczy mi, by się podniecić. | 1 | 2 | 3 | 4 |
| 13. | Jeśli myślę o tym, czy będę mieć orgazm, znacznie trudniej jest mi się podniecić. | 1 | 2 | 3 | 4 |
| 14. | Jeśli druga osoba naprawdę mnie pragnie seksualnie, bardzo się podniecam | 1 | 2 | 3 | 4 |
| 15. | Fantazjowanie o seksie sprawia, że szybko się podniecam. | 1 | 2 | 3 | 4 |
| 16. | Jeżeli jestem niepewny uczuć mojego partnera, trudniej jest mi się podniecić. | 1 | 2 | 3 | 4 |
| 17. | Podniecają mnie określone zapachy. | 1 | 2 | 3 | 4 |
| 18. | Często sam zapach drugiej osoby może działać na mnie podniecająco | 1 | 2 | 3 | 4 |
| 19. | Jeśli myślę o kimś dla mnie atrakcyjnym seksualnie, łatwo się podniecam. | 1 | 2 | 3 | 4 |
| 20. | Łatwo się podniecam z nowym partnerem. | 1 | 2 | 3 | 4 |
| 21. | Jeżeli widzę kogoś ubranego w seksowny sposób, łatwo się podniecam. | 1 | 2 | 3 | 4 |
| 22. | Jeśli martwię się tym, że mogę potrzebować zbyt dużo czasu by się podniecić, wpływa to negatywnie na moje podniecenie | 1 | 2 | 3 | 4 |
| 23. | By uzyskać pełne podniecenie, muszę ufać parterowi. | 1 | 2 | 3 | 4 |
| 24. | Trudno jest mi utrzymać stan podniecenia seksualnego. | 1 | 2 | 3 | 4 |
| 25. | Jeżeli jestem podniecona, nawet najdrobniejsza rzecz potrafi mnie rozproszyć tak, że tracę podniecenie. | 1 | 2 | 3 | 4 |
| 26. | Jeśli wszystko nie jest po mojej myśli, jest mi trudno się podniecić. | 1 | 2 | 3 | 4 |

**KLUCZ (SCORING)**

**Łatwość uzyskania podniecenia (Arousability**): (Q2+Q6+Q10+Q12 +Q14 +Q15)/6

**Charakterystyka partnera (Partner Characteristics**): (Q4+Q8+Q19+Q20+Q21)/5

**Dynamika dominacji seksualnej (Sexual Power Dynamics):** (Q1+Q4)/2

**Zapach (Smel)l**: (Q17+Q18)/2

**Zaniepokojenie wydolnością seksualną (Concerns about Sexual Function**): (Q5+Q13+Q22)/3

**Zdolność uzyskania i utrzymania podniecenia (Arousal Contingency):** (Q24+Q25+Q26)/3

**Znaczenie związku (Relationship Importance):** (Q7+Q9+Q11+Q16)/4

**Skala Pobudzenia Seksualnego (Sexual Excitation Scale):**
(Arousability + Partner Characteristics + Sexual Power Dynamics + Smell)/4

**Skala Hamowania Seksualnego (Sexual Inhibition Sale):**
(Concerns about Sexual Function + Arousal Contingency + Relationship Importance)/3

**UWAGI:**

Wyższy wynik oznacza większy poziom pobudzenia/hamowania
(Higher scores correspond with greater propensity)

W polskiej wersji w Skali Pobudzenia Seksualnego brak podskala “Miejsce”.
(Please note that Polish version does not have Setting subscale of Sexual Excitation Scale)
