# Supplementary material for: Sexual inhibition and sexual excitation in a sample of Polish women
Source: PLoS One. 2021 Apr 6;16(4):e0249560. doi: 10.1371/journal.pone.0249560 (PMC8023475; doi:10.1371/journal.pone.0249560)
Supplement: Attachment — Submitted filename: PLOS reviewrs comments 2021 R2.docx [file pone.0249560.s004.docx]

Comments by the Editor

Dear Editor,

Thank you for your valuable remarks. The answers are written below.

1. The manuscript has been revised by an English speaker, however, there are still some typos and mistakes (e.g., sentence structure, line 57, “self-report questionnaire”, line 88, Arousal Contingency (not Contingence), SESII-WW, line 103, refused, line 201) left. Please include another round of language revision before resubmission.

Corrected, as requested

2. Line 62: Please exchange the dated term “sexual drive” with sexual desire or sexual motivation.

Corrected, as requested

3. Line 155: Central/Eastern European

Corrected, as requested

4. Line 179: Please indicate the expected direction of the relationships

Added, as requested

5. Line 289: Needle sharing is not considered a sexual behavior per se

That was change to “or drug injection with shared needles leading to sex”

6. Line 307-309: Aggregating? Also, please reformat this statement.

Corrected, as requested

7. Line 338: The version of the R software is not the same as the packages (e.g., lavaan, psych, semtools) used for the analysis. Please include the packages used in the description.

Added, as requested

8. Line 372: SISII-W-PL?

That abraviation indicates the Polish version of the scale

Explained in the text, as requested.

9. Line 477: removed excluded

Corrected, as requested

10. Line 503: I assume that you used the Chi²-test to determine measurement invariance. However, the differences in CFI and RMSEA seem to suggest that the model is measurement invariant across education groups. Please consider using changes in these values as a basis for determining invariance.

Cleared, as requested.

11. Line 578: the discussion is quite lengthy and I suggest condensing it to a max. of seven pages to improve readability.

That you for that suggestion. Some changes was made, as suggested. However, we think that further condensing the discussion would affect the quality of this detailed analysis of obtained results. Now the Discussion section has six pages plus 1.5 for Clinical Implication, one for Limitation and 0.5 for Conclusions. We hope that now it will meet your expectations.

Reviewer #1: Thank you for addressing most of the comments on my previous review, and improving the overall written style of the manuscript. Overall this seems to be a thorough validation study and will make a useful contribution to the literature. My remaining comments are minor.

Dear Reviewer,

Thank you for your valuable comments. The answer are written below.

1) For the third sentence of the introduction, instead of "they moderate mutual activities" it might be more clear to say "they mutually moderate behavior".

Corrected, as requested.

2) For the second paragraph of the introduction, can a citation be provided for Gray's work on general inhibition and excitation?

Added, as suggested

3) For the rationale and hypotheses of the current study, I still think that it would better to discuss some of the material brought up in the discussion (e.g., the consideration of the impact of Catholic and post-Soviet sociocultural contexts) in the introduction instead. This is because 1) you are making a case that the East/Central European context is important to consider despite previous studies in primarily West European countries, and 2) you are specifically hypothesizing differences due to sociocultural factors. Therefore, it may be best to provide some context for these sociocultural differences when they are brought up in the introduction. And it would allow you to save some space in the discussion as well since you won't need to go over the information in detail there anymore.

Modified as suggested.

4) For the Cronbach's alphas of other measures in the current study, I still think that it would be more useful to report the numbers for the current data. Or, at least perhaps a direct statement saying that all alphas were comparable to the reported literature values would be useful.

All Cronbach's alphas for the current data are presented in Table 5

5) For the discussion of association of lower scores on both SE & SI with age in the current sample, did the authors consider a possible response bias or some other cohort effect that might differentially impact the reporting, awareness, or participation of younger versus older Polish women?

Yes, we analyzed that in context of age and social desirability. However, we have not found any differences between different age-groups (especially younger vs older). It might be speculated that if the age range in the current study was wider (e.g. up to 80 years old) the association could be different. But that need further studies. Social desirability did not have any influence either.

6) For the sentence that begins on line 749, it refers to "psychotherapy with 5-HTPA1 receptor agonist...", should this be pharmacotherapy? As well, for this part of the discussion, would psychotherapy for anxiety about sexuality be indicated for worries about imperfect sexual circumstances?

Is should be: Prior research suggests that psychotherapy combined with 5-hydroxytryptamine1A receptor agonist plus Phosphodiesterase-5 inhibitors (PDE-5i) could be a potential therapeutic option [31] for women with high Arousal Continence (i.e., worries that circumstances of sexual activity will not be “perfect”) and high Concerns about Sexual Function (i.e., performance anxiety). That sentence was changed accordingly.

7) For the paragraph starting on 765, could the authors clarify how the SESII-W scale might be used in order to facilitate education, reduce anxiety, etc.?

That sentences were changed to: . Filling in the scale may therefore have an educational function – while reading the questions/statements women may learn about sexual responses, possible reasons for performance anxiety, and may acknowledge and accept changes in sexual responses capacity in different life stage. The SE scale, in the same mechanism, may also motivate women to seek sexually exciting situations and individual experiences to increase sexual responses in different models, not only the linear models [16], and irrespective of potential limitations (e.g. physical limitations)
